# Angiotensin-Converting Enzyme Inhibitor-Induced Angioedema: A Case Report With a Review of Management Options

**DOI:** 10.7759/cureus.40320

**Published:** 2023-06-12

**Authors:** Muhammad Atif Ameer, Javaria Mushtaq, Haroon Chaudhry, Nimi Patel, Somia Ilyas Khan

**Affiliations:** 1 Medicine, Punjab Rangers Teaching Hospital, Lahore, PAK; 2 Cell Biology and Neuroscience, Rowan University, Stratford, USA; 3 Internal Medicine, Suburban Community Hospital, East Norriton, USA; 4 Pediatric Surgery, Shaikh Zayed Medical Complex, Lahore, PAK

**Keywords:** lisinopril, angiotensin-converting enzyme inhibitors, angioedema, hypertension, hypertension and therapy, drug allergy, drug-induced angioedema, side effects of lisinopril

## Abstract

Angiotensin-converting enzyme inhibitors (ACEIs) are widely used for heart failure, renal failure, diabetic nephropathy, stroke, arterial hypertension, and a number of other cardiovascular or related conditions. ACEI-induced angioedema is a rare entity but can result in life-threatening emergencies. It mainly occurs in patients starting on ACEI as an antihypertensive. We present a case of lisinopril-induced angioedema in an African American patient managed in the emergency department. After appropriate evaluation, the patient was declared safe to be observed in the emergency department. Intubation was not performed. Early identification of angioedema is paramount, and emergency physicians should maintain airways or intubate such patients if indicated. There should be a high level of suspicion of angioedema in patients taking ACEIs if they present with symptoms of respiratory compromise.

## Introduction

Angiotensin-converting enzyme inhibitors (ACEI) are commonly used antihypertensive medications estimated to be used by millions of hypertensive patients globally [1]. The three main indications for ACEI are coronary artery disease, renal disease, and heart failure. ACEI-induced angioedema incidence ranges from 0.1% to 0.7%. It has predilections in elderly individuals, females, and the African American population with a prior history of cutaneous drug eruptions, allergy reactions, and patients on immunomodulatory agents [2]. ACEI-induced angioedema in genetically predisposed individuals is caused by kallikrein activation leading to elevated levels of bradykinin, substance P, and other inflammatory mediators. These inflammatory markers are responsible for causing significant vasodilatation leading to hypotension. Clinical features range from angioedema of the face, lips, and oral mucosa to laryngeal and sub-glottic edema, hoarseness of voice, inspiratory stridor, acute tongue edema leading to pharyngeal tonsils, and uvula leading to airway compromise and asphyxiation [3]. Treatment includes removing offending agent exposure, immediate preservation of the airway, intravenous steroids, histamine receptor antagonists, and IV epinephrine once the patient is stable. Newer and improved treatments include bradykinin receptor antagonists, kallikrein production inhibitors, fresh frozen plasma, and C1 inhibitor concentrate [4]. We present a case of a middle-aged African American male with a history of ACEI-induced angioedema, who presented again with similar features after accidental re-exposure.

## Case presentation

A 48-year-old African American male presented to the emergency department with complaints of difficulty and painful swallowing along with lip swelling for the past six hours. He had a medical history of diverticulitis, gastrointestinal bleeding associated with acute gastritis, hyperlipidemia, hypertension, and spinal stenosis with sciatic pain. The patient takes atorvastatin, bupropion, metformin, cyclobenzaprine, lisinopril, and meloxicam.

**Figure 1:**
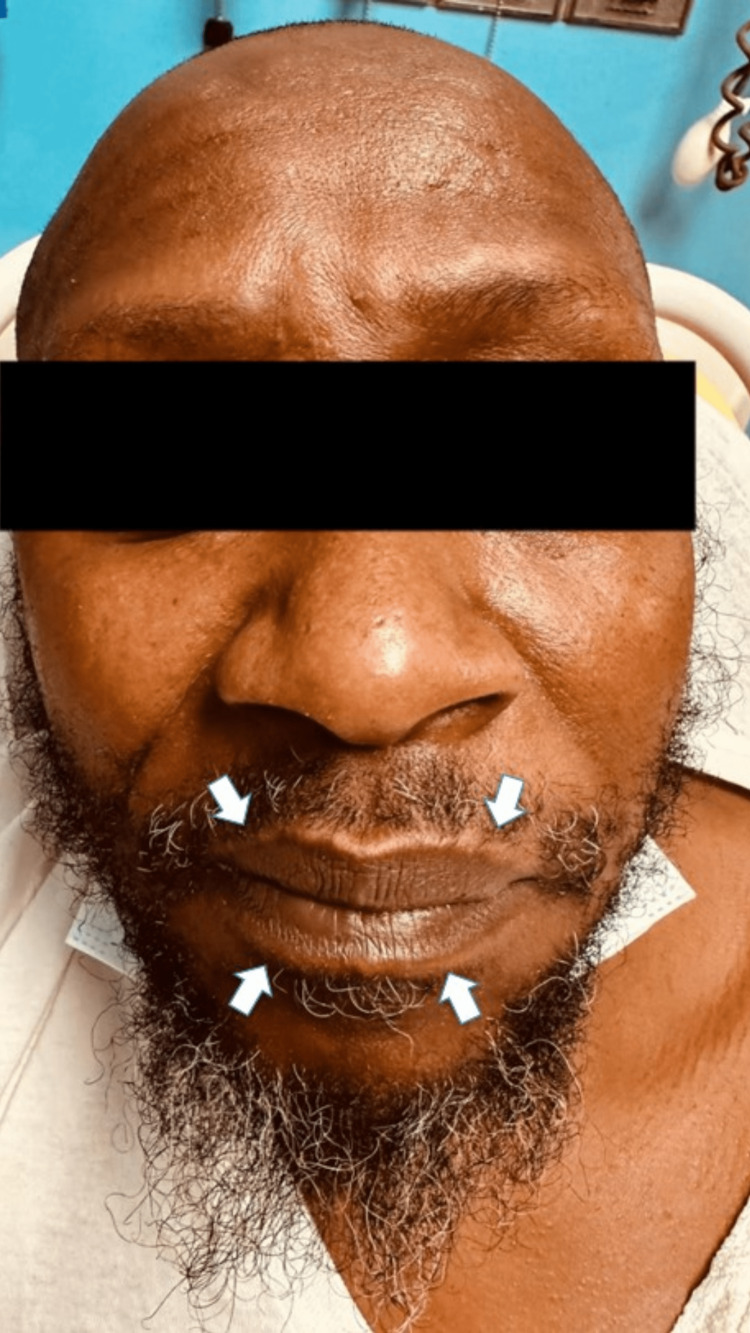
Asymmetric labial angioedema (white arrows)

**Figure 2:**
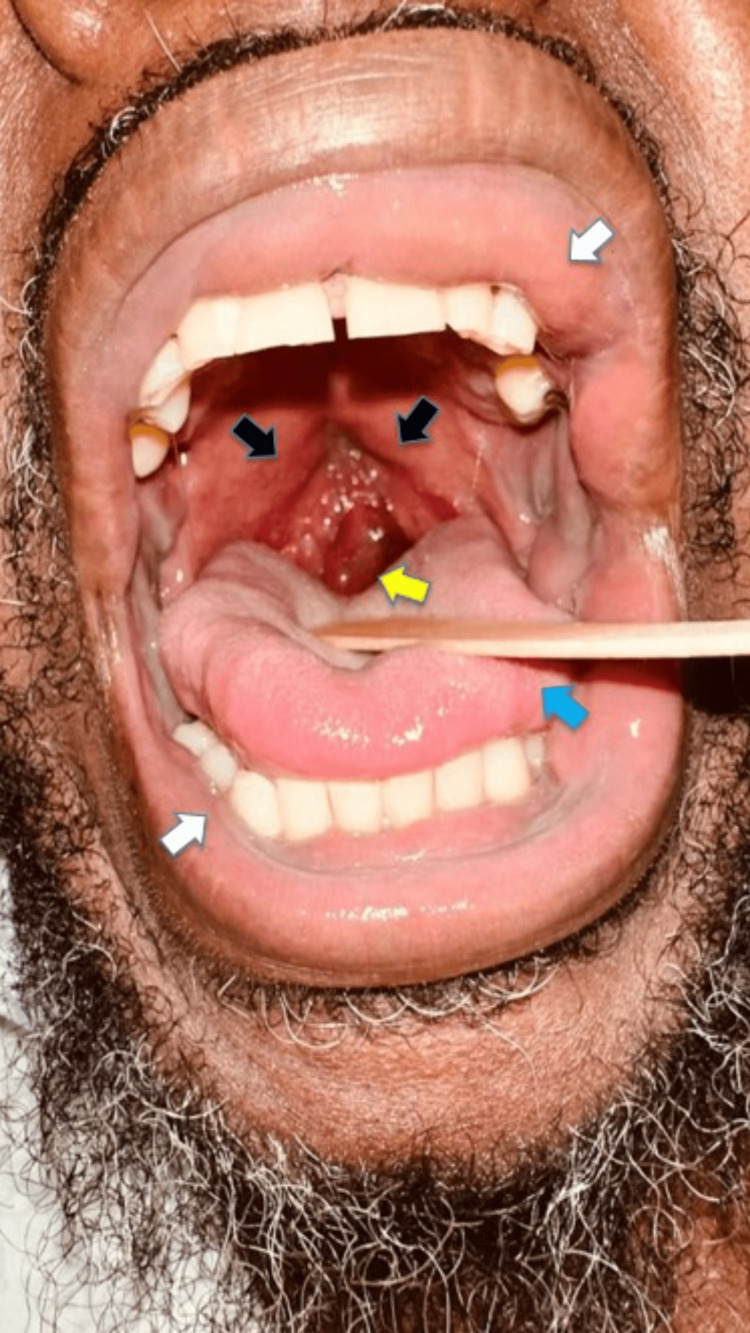
Labial, sub-labial, and gingival edema (white arrows), edematous glossopharyngeal arch and hyperemic swollen palatine tonsils (black arrows), injected and displaced edematous uvula (yellow arrow), and lingual edema (blue arrow)

On presentation, his blood pressure was 126/84 mmHg, heart rate was 103 bpm, temperature was 97.3°F, and respiratory rate was 15/minute with oxygen saturation of 95% on room air. The patient was visibly anxious with mild distress. The head was normocephalic and atraumatic on physical examination, with no apparent deformities of both ears, rhinorrhea, or nasal swelling. The patient had labial, sublabial mucosal (Figure 1), and gingival edema (white arrows), edematous glossopharyngeal arch, and hyperemic edema palatine tonsils (black arrows), injected and displaced edematous uvula (yellow arrow), and lingual edema (blue arrow). He had moderate cervical lymphadenopathy and swelling of the posterior pharynx and tonsils (Figure 2). Cardiovascular, pulmonary, gastrointestinal, and neurological exam was unremarkable.

The initial symptoms were significant for dysphagia, odynophagia, and palatal angioedema that started approximately 48 hours prior to the presentation. Additionally, the patient reported a feeling of burning sensation, predominantly in the throat and palate, exacerbated by eating or drinking. The patient denied drooling, shortness of breath, facial hyperthermia, or hyperemia. The patient reported allergies to iodine and certain medications but could not recall the specific names.

The patient was started on lisinopril five days ago for his uncontrolled hypertension. He had a similar episode a year ago when he began lisinopril. He did not seek any medical attention for it but discontinued lisinopril. He did not take this medication until recently when he was found to have uncontrolled hypertension and restarted on lisinopril.

The patient's comprehensive metabolic panel and complete blood count are given in Table 1 and Table 2, respectively. Urine drug screen and urinalysis were negative for any significant findings.

**Table 1:** Comprehensive metabolic panel

Lab	Patient value	Normal range
Sodium (Na)	138 mEq/L	135-145 mEq/L
Potassium (K)	4 mEq/L	3.5-5.0 mEq/L
Chloride (CL)	101 mEq/L	96-106 mEq/L
Bicarbonate (HCO3)	28.4 mEq/L	23–29 mEq/L
Glucose	141 mg/dL	70-100 mg/dL
Bilirubin (total)	0.3 mg/dL	0.1–1.2 mg/dL
Blood urea nitrogen (BUN)	14 mg/dL	6-24 mg/dL
Creatinine (Cr)	0.9 mg/dL	0.5-1.0 mg/dL
Aspartate aminotransferase (AST)	21 U/L	8-33 U/L
Alanine aminotransferase (ALT)	38 U/L	7-56 U/L
Alkaline phosphatase (Alk Phos)	119 U/L	30-120 U/L
Magnesium (Mg)	2 mEq/L	1.3-2.1 mEq/L
Calcium (Ca)	8.9 mEq/L	8.5-10.2 mEq/L

**Table 2:** Complete blood count

Lab	Patient value	Normal range
White blood cell (WBC)	7.6 x 109/L	4.5- 11.0 x 109/L
Red blood cells (RBC)	5.7 cells per mcL	4.7-6.1 cells per mcL
Hemoglobin (Hgb)	14.2 g/dL	13.8-17.2 g/dL
Hematocrit (Hct)	44.60%	41%-50%
Platelets	350 x 109/L	150-400 x 109/L
Mean corpuscular volume (MCV)	77.2 fl	80-100 fl
Mean corpuscular hemoglobin (MCH)	24.6 pg	27.5-33.2 pg
Mean corpuscular hemoglobin concentration (MCHC)	31.9 g /dL	32-36 g /dL
Red blood cell distribution with (RDW)	15.80%	11.8-14.5%
Mean platelets volume (MPV)	7.9 fL	7.2-11.7 fL

The patient was managed with dexamethasone 10 mg IV, diphenhydramine 50 mg IV, and famotidine 20 mg IV. Lisinopril was discontinued from the patient's regimen, and the patient was initiated on hydrochlorothiazide for his hypertension management.

## Discussion

Angioedema is a medical condition characterized by non-dependent, non-pitting swelling of deep dermal layers and subcutaneous and submucosal tissue [5]. It often occurs in different body locations, including the face, lips, tongue, throat, supraglottic region, and, less frequently, the subglottic region [6]. Mucosal layers of the gastrointestinal tract and genitalia are other important sites that angioedema can affect. Angioedema was initially defined by J.L. Milton in 1876. The illness was first given the label angioneurotic edema by Quincke in 1882 [7]. It is a self-limited condition but becomes potentially life-threatening when it involves the upper airway. Affected areas usually are skin-colored, ill-defined, and non-pitting. Nevertheless, it can also happen in conjunction with urticaria [8].

Angioedema may be classified as hereditary or acquired. Hereditary angioedema (HAE) is a rare autosomal dominant disorder. It occurs due to deficiency or dysfunction of complement C1 esterase inhibitor (C1-INH). It often manifests in early childhood as periodic, nonpruritic angioedema, developing usually over two to three days. HAE can endanger the airway due to laryngeal edema, most commonly occurring in the gastrointestinal system and extremities. The pathophysiology of HAE is associated with enormously increased levels of bradykinin in the blood, which escalates vascular permeability. There are multiple causes of acquired angioedema, which consist of acquired C1-INH deficiency due to the production of an autoantibody to C1-INH, inhalants, and idiopathic [9].

When compared to the Caucasian population, the incidence of ACEI-related angioedema is roughly three times higher in African American patients, with increased risk in elderly patients (>65 years of age) who have a history of drug rash and seasonal allergies. Additionally, the American Society of Anesthesiologists (ASA) class III-V, tobacco consumption history, cardiovascular problems, Hispanic race, and swelling in the deeper tissues of the aerodigestive tract as initial manifestations are risk factors for severe presentation. Additionally, the likelihood of it happening in the first week of therapy is 14 times higher [10].

ACEI is one of the most prevalent causes of acquired angioedema, making up about 25-39% of cases. Certain other drugs have been documented as the culprit of acquired angioedema, including angiotensin II receptor antagonists, aspirin, non-steroidal anti-inflammatory drugs (NSAIDs), fibrinolytic, statins, proton pump inhibitors, and selective serotonin reuptake inhibitors. Angioedema due to ACEI has grown significantly over the past several years and is currently used by an estimated 40 million individuals globally.

Angioedema has been observed shortly after the invention of ACEI, which suggested that the underlying mechanism was heightened kinin effects brought on by kininase II inhibition [7]. Pre-kallikrein, which is transformed into kallikrein, and high molecular weight kininogen are the components of the bradykinin pathway. Both hereditary and acquired angioedema are forms of bradykinin-mediated angioedema caused by a lack of or dysfunction in the factor C1 inhibitor, which causes an increase in kallikrein production and its conversion to bradykinin activity. Angioedema has been observed to occur with the use of all ACEI, and it is regarded as a class-related side effect. Inhibition of angiotensin-converting enzyme (ACE) prevents the conversion of angiotensin and lowers the degradation of the powerful vasoactive peptide bradykinin, which ACE destroys. As a result, it has been hypothesized (with scant scientific support) that ACEIs cause angioedema via enhancing bradykinin production [11].

Bradykinin and substance P activity increases tissue permeability and increases vasodilation, resulting in edema and swelling of the tissues. The face and airway are frequently affected by ACEI-induced angioedema. Up to 10% of patients experience airway compromise, which is occasionally fatal [7]. Additionally, a visceral component can impact the mucosa of the entire intestinal system and appear as nausea, vomiting, diarrhea, and abdominal cramps. Between 0.1% and 0.5% of patients taking the medication may experience angioedema caused by an ACEI [7]. There have been reports of fatal ACEI-induced angioedema, despite the fact that it is less common than life-threatening angioedema. Drug-induced angioedema is most frequently caused by ACEIs [7].

Airway management, which includes diligent monitoring and endotracheal intubation or surgical airway, is crucial. It is essential to stop the inciting agent promptly. Even though ACEI-induced angioedema symptoms should go away 48 to 72 hours after they first appear, the medicine should never be resumed because of the frequency of reoccurrence [10].

It is believed that a disruption in the bradykinin pathway causes ACEI-induced angioedema, and more recent drugs have proven helpful in treating this potentially fatal condition. Icatibant is a specific antagonist of bradykinin B2. It is being used to treat HAE and when compared to conventional steroid and antihistamine therapy, it shows comparable relief after a single dose [12]. In rare instances, ACEI-induced angioedema can also improve from purified C1 concentrate. Ecallantide has not always been effective in treating ACEI-induced angioedema, even though it has been used to treat genetic angioedema [12]. Antihistamines, epinephrine, and steroids are the standard treatments for angioedema. However, despite their use, there is limited proof of their effectiveness, specifically in ACEI-induced angioedema. It can be challenging to diagnose the pathology correctly, but it may be essential to starting the right treatment [13].

## Conclusions

ACEI-induced angioedema is a rare adverse effect of one of the most commonly used antihypertensive medications. It can result in life-threatening complications and/or airway obstruction. Whenever such patients are encountered, this differential should be kept in mind. In addition to protecting the airway, the inciting agent should be promptly discontinued. Sometimes, these patients may not initially respond to steroids, epinephrine, or antihistamines, so the airway should be secured via endotracheal intubation or surgically if a respiratory compromise is suspected. Purified C1, icatibant, and sometimes fresh frozen plasma can also be used if the condition is unresponsive to the initial treatment.
